# Sanji Peiyuan decoction combined with dydrogesterone in the treatment of massive subchorionic hematoma: A case report

**DOI:** 10.1097/MD.0000000000036382

**Published:** 2023-12-08

**Authors:** Yi Yang, Huiling Zheng, Huijun Ye, Yunxia Lin, Jiali Liu, Ruilan Li, Lihua Jin

**Affiliations:** a Department of Gynaecology and Obstetrics, The Second Affiliated Hospital of Zhejiang Chinese Medical University, Hangzhou, China.

**Keywords:** Breus’ more, case report, hydrogesterone, massive subchoric thrombohema, sanji peiyuan decoction, subchorionic hematoma, traditional Chinese medicine treatment

## Abstract

**Rationale:**

Subchorionic Hematoma, often referred to as Bruce hematoma, can lead to serious obstetric complications such as intrauterine growth restriction and fetal death, as well as early and late pregnancy miscarriage, placental abruption, and premature rupture of membranes, posing great harm to both mother and fetus.

**Patient concerns:**

At present, Western medical treatments have not shown satisfactory results, necessitating the discovery of more effective clinical treatment methods.

**Diagnoses:**

Threatened miscarriage, Subchorionic hematoma, Iron deficiency anemia (mild).

**Interventions:**

Sanji Peiyuan decoction combined with dydrogesterone.

**Outcomes:**

Following 17 days of treatment with Sanji Peiyuan decoction and oral dydrogesterone tablets, the hematoma was no longer detectable by ultrasound. The patient experienced no symptoms such as abdominal pain, bloating, or vaginal bleeding. She successfully gave birth around her due date, with both the mother and child in good health.

**Lessons:**

The combination of Sanji Peiyuan decoction and oral dydrogesterone tablets shows promising clinical efficacy in treating Massive Subchorionic Hematomas. This method merits further clinical research.

## 1. Introduction

Subchorionic hematoma denotes the accumulation of blood between the chorionic membrane and the decidua basalis, stemming from their separation. Its prevalence among pregnant women ranges from 4% to 48%, and it is recognized as a significant factor influencing perinatal outcomes.^[1]^ Ultrasound usually reveals a hypoechoic or anechoic crescentic space behind the fetal membrane, which might lift the placental edge. The risk of miscarriage rises with the size of the hematoma.^[2]^ When a hematoma surpasses 1 centimeter in thickness, it classified as a massive subchorionic thrombohematoma (MTH) or Breus’ hematoma. This condition can result in grave obstetric outcomes like intrauterine growth restriction and intrauterine fetal death (IUFD), as well as miscarriages, placental abruption, and premature rupture of membranes.^[3]^ The condition threatens both mother and fetus alike. Present Western treatments do not offer satisfactory results, whereas traditional Chinese medicine offers quicker hematoma resolution. This report details the use of Sanji Peiyuan decoction, a traditional Chinese medicine, in tandem with oral dydrogesterone tablets to treat a mid-pregnancy MTH case. After a 17-day treatment course, the hematoma was fully resolved, eliminating the need for intramuscular or intravenous injections. This treatment approach is straightforward, has a brief duration, is cost-effective, and offers ease of medication administration.

The Second Affiliated Hospital of Zhejiang Chinese Medical University Ethics Commission for human research sanctioned the study, ensuring the ethical standards and integrity of the research were maintained, and the patient gave informed consent.

## 2. Case presentation

### 2.1. Clinical presentation

A Chinese female, 23 years old, was initially diagnosed on December 29, 2020 after 86 days of amenorrhea and 1 day of ultrasound detection of a subcolonal hematoma. The patient has a regular menstrual cycle of 28 days. Her last menstrual period was on October 4, 2020. After more than 30 days without menstruation and post-intercourse, she experienced light vaginal bleeding of a bright red hue. No accompanying symptoms of abdominal pain, bloating, fever, or chills were present. On external hospital examination, her level of β-chorionic gonadotropin (β-HCG) was noted as 980.50 IU/L, and progesterone was 75.30 nmol/L. An ultrasound revealed: “Possible intrauterine gestational sac measuring roughly 0.3 cm × 0.2 cm × 0.2 cm.” For fetal protection, she was prescribed Progesterone capsule 100mg taken twice daily. the vaginal bleeding had ceased, and she felt well. A follow-up ultrasound on December 1, 2020, after 57 days of amenorrhea, showed an “echogenic gestational sac of dimensions 32 mm × 30 mm × 32 mm in the uterine cavity with a visible yolk sac inside. The bud length was approximately 15mm, consistent with the gestational age, and a primitive cardiac pulsation was observed.” Due to high uterine artery blood flow, she was treated with 14 days of subcutaneous injection of 4000U qd enoxaparin and 100mg qd aspirin enteric coated tablets. On December 14, 2020, at 71 days of amenorrhea, she experienced pronounced back pain and occasional lower abdominal distension but no abdominal pain or vaginal bleeding. An ultrasound on December 29, 2020, revealed irregular dark regions in the chorionic villi and the left and posterior walls of the uterus, measuring roughly 59 mm × 54 mm × 19 mm. The diagnosis was: “Intrauterine single live fetus approximating 11w + 6d in size; subchorionic hematoma suspected.” She reported lower back pain, slight nausea, but no vomiting, abdominal pain, bloating, vaginal bleeding, fever, chills, or diarrhea. The patient has no other significant medical or known allergic history. She got married at 22 and has a healthy spouse. Her record shows 0-0-3-0, indicating she had 3 prior induced abortions. Menarche began at the age of 13, with a period of 4 to 5 days and a cycle of 28 days. The last menstrual date was October 4, 2020. The patient neither smokes nor consumes alcohol. No relevant family medical history was provided. She denies having any contact history in affected areas. T: 36.7ºC, P: 87 times/min, R: 20 times/min, BP: 100/63 mm Hg. Patient is alert, in good spirits, with normal heart and lung auscultation. Her abdomen is soft without hepatosplenomegaly, and no tenderness or rebound pain. Gynecological exam showed no external genitalia abnormalities, a smooth vagina with minimal white discharge, smooth cervix, an anteriorly placed enlarged uterus resembling 3 months gestation, and no tenderness with normal mobility. No abnormalities were detected in both adnexa. The oral examination revealed a pale-red tongue with thin, white coating and faint pulsations. November 11, 2020: Blood B-HCG: 980.50 IU/L, progesterone: 75.30 nmol/L. 2020-11-16: Blood B-HCG: 8247.53 IU/L, progesterone: 78.40 nmol/L. December 30, 2020: Blood β-HCG: 94707.0 IU/L, estradiol: 10560.0 pmol/L, progesterone > 190.80 nmol/L. December 30, 2020: Thyroid function: antithyroglobulin antibodies thyroperoxidase antibodies: normal range. December 30, 2020: Blood routine test: Hemoglobin 105g/L. On December 1, 2020, B-ultrasound was performed in an external hospital. Echogenicity of a 32mm * 30mm * 32mm gestational sac was detected in the uterine cavity, and a yolk sac was seen inside. The bud length was about 15mm, and primitive cardiac pulsation was visible. Left side of uterine artery blood flow: S/D: 30.27, PI: 3.84, RI: 0.97, right side: end diastolic blood flow reversal, imaging diagnosis: early pregnancy, intrauterine pregnancy, right uterine artery end diastolic blood flow reversal. On December 29, 2020, B-ultrasound was performed in an external hospital. A fetus was detected in the uterus, with a head to hip diameter of 52 mm and a biparietal diameter of 17 mm. The placenta was located on the anterior wall of the uterus, at level 0. The depth of amniotic fluid was about 30 mm, and the heart rate was about 169 beats per minute. The thickness of the neck NT is approximately 1.00 mm. Irregular dark areas can be seen between the chorionic villi and the left and posterior walls of the mother uterus, ranging from approximately 59 mm * 54 mm * 19 mm, with dense peristalsis of light spots. Bilateral uterine artery resistance index: left S/D: 4.90, PI: 1.98, RI: 0.80, right S/D: 4.01, PI: 1.70, RI: 0.75. Imaging diagnosis: Intrauterine single live fetus, equivalent to 11w + 6d in size; Subchorionic hematoma is considered. The Final Diagnosis: Threatened miscarriage, Subchorionic hematoma, Iron deficiency anemia (mild).

### 2.2. Interventional procedure

According to TCM theory, we determined to give her Oral intake of the Chinese medicinal concoction, Sanji Peiyuan decoction: 6g of Sanji powder (swallowed), 6g of Baiji, 30g of Codonopsis pilosula, 15g of stir fried Atractylodes macrocephala, 15g of raw white peony, 15g of goji berry, 15g of dodder seed, 15g of ligustrum lucidum, 10g of baicalin charcoal, 15g of ramie root, 30g of Eucommia ulmoides, 30g of mistletoe, 15g of yam, 9g of sorghum, and 10g of Zhuru. Seven doses of the above drugs were decocted in water, totaling 400ml, 200ml in the morning and afternoon, and taken warm. For other treatment, Dydrogesterone 10mg taken twice daily for fetal protection, and protein succinate iron oral solution, 15ml daily, to treat iron-deficiency anemia.

### 2.3. Follow-up and patient perspective

**Figure 1.**
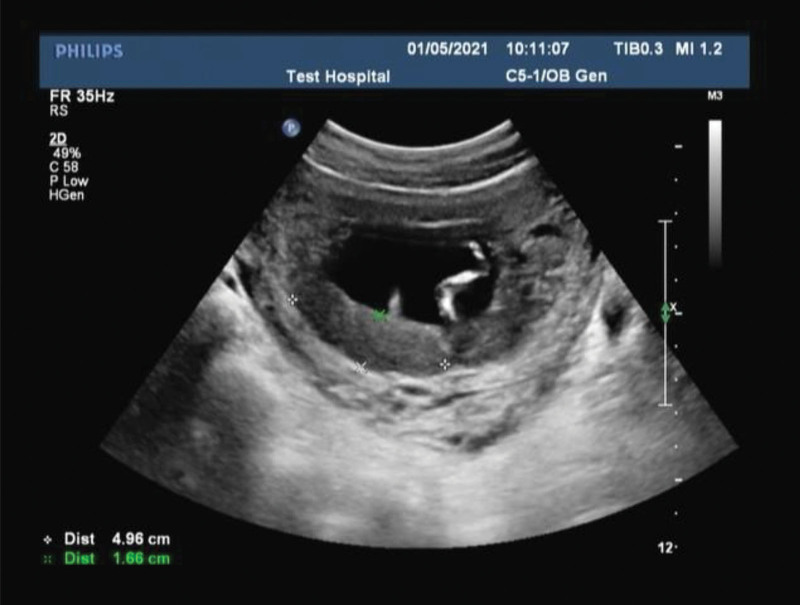
January 5, 2021: Ultrasound showed a hypoechoic region at the placenta lower edge, measuring 5.0 cm × 1.7 cm, with visible fluid movement within.

January 5, 2021: The patient reported alleviated back pain and noted no symptoms of abdominal pain, vaginal bleeding, or other discomfort such as nausea and vomiting. Ultrasound findings from our hospital concerning the fetus and its appendages revealed: fetal position as uncertain; head to hip diameter at 6.3cm; placenta observed on the right wall, left wall, and anterior wall; Gr at 0 level. A hypoechoic area of dimensions 5.0cm × 1.7cm was visible at the lower edge of the placenta with fluid flow inside. The placenta lower edge extended to the cervical opening. Amniotic fluid was measured at approximately 3.0cm deep. The fetal heart rate was consistent at 158 beats/minute, and fetal movements were detectable (Fig. 1).

January 6, 2021: Lab tests indicated Blood β-HCG levels at 94707.0IU/L, estradiol levels at 10560.0pmol/L, and progesterone > 190.80nmol/L. The patient was advised to continue with Sanji Peiyuan decoction and oral dydrogesterone tablets.

**Figure 2.**
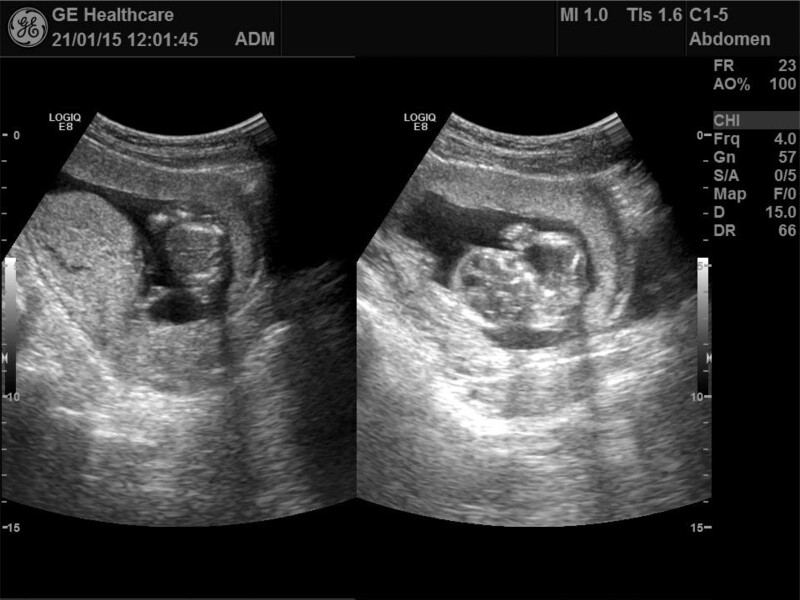
January 15, 2021: B-ultrasound displayed the resolution of the previously observed subclonial hematoma.

January 15, 2021: On the follow-up, the patient felt well without symptoms of lower back pain, vaginal bleeding, or other discomforts. A subsequent ultrasound showed: uncertain fetal position; biparietal diameter of 2.5cm; femur length of 1.3cm; dominant anterior wall of the placenta; Gr 0 grade; and amniotic fluid depth at 3.5cm. Fetal heart rate was 166 beats/minute, and its rhythm was regular. The prior subdural hematoma had completely resolved. The treatment was deemed successful (Fig. 2).

July 10, 2021: A healthy female infant was delivered vaginally, achieving Apgar scores of 10 at both 1 and 5 minutes post-birth. Post-delivery examinations found the placenta and fetal membranes to be normal. A 2-year follow-up confirmed both mother and child to be in good health.

## 3. Discussion

Giant chorionic hematoma (MTH), also known as Bruce hematoma, is a rare complication of pregnancy. It was first described by Breus in 1892.Its initial description dates back to 1892 by Breus. Despite its rare occurrence, with an incidence rate between 0.03%–0.08%,^[4,5]^ the associated risks are considerable, often leading to poor prognosis and being a primary reason for fetal mortality.^[6]^ Multiple factors contribute to MTH, including prenatal hospitalization, abortion, blood transfusion, severe fetal growth restriction, intrauterine fetal death, premature delivery, premature rupture of membranes, placental abruption, oligohydramnios, pre-eclampsia, diffuse choriohemosiderosis, and neonatal lung incidence rate.^[7,8]^ The diagnosis of MTH mainly includes the following 2 aspects: 1 cm thick hematoma involving the chorionic plate; Widely (50% or more) involving the surface of the fetal placenta.^[9]^ The causes of MTH and subchorionic hematoma are consistent, and it is generally believed that 80% of them are caused by embryonic genetic abnormalities.^[10]^ In addition, they may also be related to the following factors: abnormal coagulation function, immune and endocrine disorders, obesity, recurrent induced abortion, hypertension, and/or the use of aspirin or other anticoagulants, among which immune factors are the most closely related.^[11,12]^ The patient in this case had 3 induced abortions and used aspirin enteric coated tablets and enoxaparin needles in the early treatment, both of which are susceptible factors for the occurrence of MTH.

Traditional Chinese medicine posits that MTH patients predominantly suffer due to spleen and kidney deficiency, blood stasis and internal obstruction. These conditions represent the etiology and pathogenesis arising from a mix of deficiency and excess. A deficiency in kidney qi cannot stabilize fetal cells. If the spleen and stomach are compromised and lack the biochemical sources of qi and blood, the Chong Ren remains unstable, making it impossible to sustain the fetus. Blood facilitates circulation; when there internal obstruction and blood stasis, which interrupts circulation, the fetus becomes malnourished. If blood isn’t expelled from the uterine cavity promptly, it results in blood accumulation there. Hence, the therapeutic approach should fortify the body natural defenses while also countering harmful factors: it should strengthen the kidney and spleen, enhance blood production, and disperse blood stasis to protect the fetus. As recorded in our medical annals, the Sanji Peiyuan decoction serves as a tried-and-tested formula to treat chorionic hematomas. It has also demonstrated efficacy against MTH. Within this formula, Sanqi powder helps disperse blood stasis, staunch bleeding, and reduce hematomas. Baiji combination aids in stemming bleeding and reducing swelling, proving crucial in treating subclonial hematomas. Ingredients like Goji berries, Ligustrum lucidum, dodder seeds, salted Eucommia ulmoides, mistletoe, and ramie roots fortify the kidneys and prevent miscarriage. Meanwhile, stir-fried Atractylodes macrocephala, Codonopsis pilosula, Chinese yam, Yangchun sand, and Perilla frutescens bolster the spleen, nourish qi and blood, and assist absorption. Elements like raw charcoal, Scutellaria baicalensis charcoal, mulberry leaves, and Zhuru help in cooling, preventing miscarriages, and reducing bleeding. White peony plays a role in calming the liver, nourishing yin, and alleviating pain. Collectively, this formula is designed to enhance kidney function, invigorate the spleen, boost qi, enrich blood, stop bleeding, reduce swelling, and nurture the fetus.

Some researchers have pointed out that MTH is actually an immune vasculitis within decidual blood vessels, with its emergence tied to immune irregularities in the mother or at the maternal-fetal boundary. The equilibrium between the 2 factors is pivotal for sustaining pregnancy, and disturbances primarily stem from autoimmune and alloimmune abnormalities. Autoimmunity mainly includes anticardiolipin antibodies and antibodies β Abnormalities such as 2 glycoprotein antibodies and lupus anticoagulants: Studies have found that the titers of these 3 antibodies in MTH patients are significantly higher than those in normal pregnant patients, indicating that the presence of autoantibodies and pre thrombotic state can disrupt the stability of the mother fetal interface, leading to decidual immune vasculitis, intravascular microthrombosis, decidual vascular rupture, abnormal invasion of the trophoblast, and rebleeding after ischemia-reperfusion.^[11]^ Experimental studies have shown that Panax notoginseng has the effects of shortening clotting time, increasing platelet production, increasing platelet aggregation, improving clotting ability, and repairing the endometrium. Endometrial tissue factor is involved in the process of uterine bleeding. Panax notoginseng can increase blood fibrinolysis and coagulation function by inhibiting tissue factor, thereby achieving the goal of hemostasis^[13]^; The compound components of Panax notoginseng can inhibit the expression of matrix metalloproteinase 1 and matrix metalloproteinase 9 in the endometrium of uterine inflammatory bleeding; The ginsenosides of Panax notoginseng have the effect of promoting the increase of cAMP in platelets and inhibiting the production of thromboxane A2.^[13]^

In addition, MTH correlation with alloimmune abnormalities is evident, like the Th1/Th2 cytokine imbalance: Th1 type cytokine IFN in such patients-γ. The levels were significantly higher in women with normal pregnancy, TNF-a and IL-2 were significantly higher in the normal pregnancy group, while IL-4 and IL-10 were significantly lower in the control group. IFN-γ It can activate the endothelial cells of decidual blood vessels to release prothrombin, catalyzing the production of prothrombin into thrombin. Active thrombin promotes coagulation in decidual blood vessels, blood stasis, distal vascular ischemia and necrosis, leading to bleeding and hematoma formation.^[2]^ Th2 cytokines such as IL-10 can inhibit the action of Th1 cytokines, thereby preventing the occurrence of MTH. Integrins of the adhesion molecule family on CD56+, CD16-NK cells, and CD56+, CD16-NK + cell surfaces in peripheral blood of MTH patients α. The expression of Panax notoginseng saponins is significantly lower than that of non MTH patients, and Qian Xudai et al^[13]^ believe that Panax notoginseng saponins have the effect of promoting CD34 + cell proliferation. Additionally, specific traditional Chinese medicines, like Shoutai Wan and Zhuyun III, can decrease the chances of pregnancy loss by modulating the glycolysis equilibrium at the maternal-fetal interface in kidney deficiency threatened abortion model mice.^[14]^ For example, Shoutai Pill (composed of Sangsheng, Cuscuta, Dipsacus, ass hide glue) can regulate the expression of various proteins in decidua tissue of mice with recurrent abortion, and regulate the main signal pathways and biological processes of patients with recurrent abortion: regulate glucose metabolism and insulin resistance in decidua tissue; Regulating the VEGF signaling pathway; Improving hypoxia levels and oxidative stress in decidual tissue: Improving pre thrombotic state; Regulating the estrogen signaling pathway; Negative regulation of cell apoptosis in decidual tissue; Improving immune disorders: positively regulating T cell mediated cytotoxicity, T cell receptor signaling pathways, etc.^[14]^ Traditional Chinese medicine Zhuyun No.3 (Cuscuta chinensis, Cinnamomum cassia, Fangfeng, Dipsacus, Astragalus membranaceus, and Codonopsis pilosula) can inhibit the secretion of Th1 cytokines, promote the secretion of Th2 cytokines, restore the pathological metastasis of Th1/Th2 balance, increase serum progesterone and β-HCG concentration.^[15]^

Dydrogesterone, a member of the “progestin” class, is a stereoisomer of natural progesterone.^[16]^ It exhibits characteristics akin to natural progesterone, boasts a strong affinity for progesterone receptors, and is highly bioavailable post-oral consumption. It doesn’t exert any androgenic or anti-androgenic effects on the fetus, thus eliminating risks of fetal genital abnormalities.^[17]^ Research by Pelinescu Onciul D suggests that Dhydrogenesterone has a significant immune regulatory effect in maintaining Th2 cytokine balance, altering Th1/Th2 balance, increasing the number of peripheral progesterone receptors, and decidual lymphocytes (CD56 þ) And stimulating progesterone blockade factor (PIBF) significantly increases the success rate of fetal protection compared to micronized progesterone, making it a good choice for preventing miscarriage in women with subchorionic bleeding.^[18]^

From our clinical observations, the combination of Sanji Peiyuan decoction and dydrogesterone tablets presents a straightforward, cost-effective, minimally invasive, and effective remedy for MTH. Nevertheless, given the rarity of MTH and limited clinical cases, this discussion remains a case report. More expansive clinical studies with larger samples are imperative.

## Author contributions

**Data curation:** Huiling Zheng.

**Formal analysis:** Jiali Liu.

**Investigation:** Ruilan Li.

**Methodology:** Huijun Ye.

**Resources:** Yunxia Lin.

**Supervision:** Yi Yang.

**Writing – original draft:** Lihua Jin, Yi Yang.
